# Supplementary material for: Genomic basis of insularity and ecological divergence in barn owls (Tyto alba) of the Canary Islands
Source: Heredity (Edinb). 2022 Sep 29;129(5):281–94. doi: 10.1038/s41437-022-00562-w (PMC9613907; doi:10.1038/s41437-022-00562-w)
Supplement: Supplementary file 1 — Supplementary Material [file 41437_2022_562_MOESM1_ESM.pdf]

Supporting Information

Insularity and ecological divergence in barn owls (*Tyto alba*) of the Canary Islands

Tristan Cumer, Ana Paula Machado, Felipe Siverio, Sidi Imad Cherkaoui, Inês Roque, Rui Lourenço, Motti Charter, Alexandre Roulin, Jérôme Goudet

Contents

Supplementary Tables 2
- Sup. Table 1 2
- Sup. Table 2 3
- Sup. Table 3 4
- Sup. Table 4 6

Supplementary Figures 7
- Sup. Fig. 1 7
- Sup. Fig. 2 7
- Sup. Fig. 3 8
- Sup. Fig. 4 9
- Sup. Fig. 5 9
- Sup. Fig. 6 10
- Sup. Fig. 7 11
- Sup. Fig. 8 12
- Sup. Fig. 9 12
- Sup. Fig. 10 13
- Sup. Fig. 11 13
- Sup. Fig. 12 14

Supplementary References 15

Supplementary Tables

Sup. Table 1 – Description of barn owl samples used in this study.

#	Pop	ID	Location	Lat / long	Year	Tissue	Sex	Ref
1	East Canary	EC01	Lanzarote	29.00 / -13.62	2010	muscle	Female	1
2	East Canary	EC02	Lanzarote	28.97 / -13.67	2010	muscle	Female	1
3	East Canary	EC03	Lanzarote	29.07 / -13.48	2012	muscle	Female	1
4	East Canary	EC04	Lanzarote	28.96 / -13.65	2012	muscle	Female	1
5	East Canary	EC05	Lanzarote	29.01 / -13.55	2012	muscle	Male	1
6	East Canary	EC06	Lanzarote	28.91 / -13.78	2012	muscle	Male	1
7	East Canary	EC07	Lanzarote	29.07 / -13.48	2007	feather	Male	1
8	East Canary	EC08	Lanzarote	28.91 / -13.78	2008	feather	Female	1
9	East Canary	EC09	Fuerteventura	28.61 / -13.93	2007	feather	Male	1
10	East Canary	EC10	Fuerteventura	28.44 / -13.87	2007	feather	Female	1
11	Morocco	MO01	Mechra Bel Ksiri	34.57 / -5.94	2019	blood	Female	1
12	Morocco	MO02	Bouzinka	33.72 / -7.22	2019	blood	Female	1
13	Morocco	MO03	Rabat	33.96 / -6.89	2019	skin	Male	1
14	West Canary	WC01	Tenerife	28.33 / -16.85	1905	muscle	Male	2
15	West Canary	WC02	Tenerife	28.18 / -16.48	2003	muscle	Female	2
16	West Canary	WC03	Tenerife	28.31 / -16.41	2003	muscle	Female	2
17	West Canary	WC04	Tenerife	28.10 / -16.62	2003	muscle	Male	2
18	West Canary	WC05	Tenerife	28.33 / -16.4	2005	muscle	Male	2
19	West Canary	WC06	Tenerife	28.33 / -16.38	2005	muscle	Female	2
20	West Canary	WC07	Tenerife	28.44 / -16.48	2006	muscle	Male	2
21	West Canary	WC08	Tenerife	28.48 / -16.35	2006	muscle	Male	2
22	West Canary	WC09	Tenerife	28.39 / -16.65	2006	muscle	Male	2
23	Portugal	PT01	Pombal	39.95 / -8.72	2013	feather	Female	3
24	Portugal	PT02	Coruche	38.83 / -8.8	2013	feather	Male	3
25	Portugal	PT03	Évora	38.45 / -8.14	2013	feather	Male	3
26	Portugal	PT04	Coruche	38.87 / -8.84	2012	feather	Female	3
27	Portugal	PT05	Nazaré	39.61 / -8.98	2013	feather	Female	3
28	Portugal	PT06	Porto de Mós	39.55 / -8.81	2013	feather	Female	3
29	Portugal	PT07	Setúbal	38.60 / -8.65	2012	feather	Female	3
30	Portugal	PT08	Fátima	39.64 / -8.72	2013	feather	Female	3
31	Portugal	PT09	Santarém	39.42 / -8.85	2013	feather	Male	3
32	Israel	IS01	Lachish	31.63 / 34.85	2005	blood	Female	2
33	Israel	IS02	Beit Shean	32.47 / 35.5	2005	blood	Male	2
34	Israel	IS03	Hula	33.13 / 35.62	2005	blood	Female	2
35	Israel	IS04	Beit Shean	32.45 / 35.42	2005	blood	Female	2
36	Israel	IS05	Beit Shean	32.47 / 35.5	2005	blood	Male	2
37	Israel	IS06	Beit Shean	32.49 / 35.55	2005	blood	Female	2
38	Israel	IS07	Beit Shean	32.47 / 35.5	2005	blood	Female	2
39	Israel	IS08	Hula	33.10 / 35.61	2005	blood	Female	2
40	Israel	IS09	Hula	33.09 / 35.62	2005	blood	Female	2
41	Syngapore	SGP	Singapore	1.35 / 103.82	2013	soft tissue	Male	3
42	USA	USA	San Diego, CA	32.72 / -117.16	2015	soft tissue	Female	3

(*1*) GenBank BioProject PRJNA727977 (sequenced in this study)

(*2*) GenBank BioProject PRJNA774943

(*3*) GenBank BioProject PRJNA700797

Sup. Table 2 – ShinyGO pathway enrichment results for genes in putatively adapted genomics regions in the Canary islands barn owl populations. Only pathways enriched by more than three genes are displayed. All listed genes are located in the haplotype-like region in Figure 2, except those marked with †.

Enrichment FDR	Genes in list	Total genes	Functional Category	Genes
0.0443	15	1164	Anatomical structure formation involved in morphogenesis	GNG5 ANXA2† F3 RORA† VAV3 CCN1 PRKACB DDAH1 WDR72† S1PR1 COL11A1 ADAM12† TGFBR3 GLMN DLC1†
0.0443	25	2785	Anatomical structure morphogenesis	PLPPR4 NEXN GNG5 ANXA2† TGFBR3 F3 CCN1 COL11A1 RORA† PALMD OLFM3 VAV3 BCAR3 PRKACB BARHL2 DDAH1 NTNG1 WDR72† S1PR1 ADAM12† FGD5† DLC1† DOCK1† GLMN USP33
0.0443	14	1077	Circulatory system development	GNG5 ANXA2† TGFBR3 F3 COL11A1 ACADM VAV3 CCN1 DDAH1 S1PR1 GLMN RORA† ADAM12† DLC1†
0.0460	12	860	Tube morphogenesis	ANXA2† F3 VAV3 CCN1 PRKACB DDAH1 S1PR1 RORA† ADAM12† TGFBR3 GLMN DLC1†
0.0486	10	603	Blood vessel morphogenesis	ANXA2† F3 VAV3 CCN1 DDAH1 S1PR1 RORA† ADAM12† TGFBR3 GLMN

Sup. Table 3 – List of genes in putatively adapted genomic regions in the Eastern Canary population, grouped per location on the barn owl genome. Associated phenotypes are provided for each gene when available. Human-specific behavioural phenotypes are not reported (for example, alcohol consumption). Genes marked with † have phenotypes related to body size and proportions, and ‡ with blood parameters.

Super-Scaffold	Gene	Phenotype	References
3	LRTM1†	Body height	(*4*, *5*)
5	LAS1L	-	
	MSN	Alopecia	(*6*)
	HEPH	Alopecia	(*6*)
	EDA2R	Alopecia	(*6*)
16	SRBD1	-	
	DAAM2†‡	Body height	(*4*, *7*)
		Platelet count	(*8*)
23	MMAA‡	Hemoglobin measurement	(*9*, *10*)
44	PITPNM2‡	Platelet count	(*11*)
		Reticulocyte count	(*10*)
		BMI	(*4*)
	ARL6IP4	-	
	OGFOD2	-	
	ABCB9†	Waist-hip ratio	(*4*)
		BMI-adjusted waist-hip ratio	(*12*)
	VPS37B†	BMI-adjusted waist-hip ratio	(*12*)
	HIP1R†‡	BMI	(*13*)
		BMI-adjusted waist-hip ratio	(*12*)
		Waist-hip ratio	(*4*)
		Body height	(*4*)
		Platelet count	(*10*)
	CCDC62†‡	BMI-adjusted waist-hip ratio	(*12*)
		Platelet count	(*10*)
	MPHOSPH9‡	Platelet count	(*9*, *10*, *14*)
	MTRFR	-	
	CDK2AP1	-	
	SBNO1‡	Lymphocyte count	(*10*)
20000042	P3H1	-	
	CLDN19	-	
	YBX1	-	
	PPIH	-	
	CCDC30‡	Systolic blood pressure	(*15*)
		Pulse pressure	(*15*, *16*)
	PPCS	-	
	PLOD1‡	Platelet count	(*14*)
	KIAA2013†	Waist-hip ratio	(*5*)
	CLCN6‡	Diastolic blood pressure	(*17*, *18*)
		Systolic blood pressure	(*4*, *17*, *18*)
		Pulse pressure	(*18*)

		Mean arterial pressure	(*19*)
		Diastolic blood pressure	(*17*, *18*, *20*, *21*)
		Systolic blood pressure	(*18*, *20–23*)
		Pulse pressure	(*18*)
	MTHFR‡	Platelet count	(*8*, *10*)
		Blood pressure	(*20*)
		Hypertension	(*24*)
		Mean corpuscular volume	(*4*, *8*, *10*)
		Erythrocyte count	(*4*, *10*)

Sup. Table 4 – List of genes in putatively adapted genomic regions in the Western Canary population, grouped per location on the barn owl genome. Associated phenotypes are provided for each gene when available. Human-specific behavioural phenotypes are not reported (for example, alcohol consumption). Genes marked with † are linked to red blood cells and haemoglobin measurements, and with ‡ to other blood parameters.

Super-Scaffold	Gene	Phenotype	References
2	WNT11	Platelet count	(*9*, *10*)
7	GRB14	Hemoglobin measurement	(*9*)
		Erythrocyte count	(*10*)
	COBLL1	BMI-adjusted waist-hip ratio	(*5*, *12*)
		Erythrocyte count	(*14*)
		Systolic blood pressure	(*4*, *14*, *16*)
8	OTUD7B	Body height	(*25*)
		BMI-adjusted waist-hip ratio	(*12*)
	MTMR11	Body height	(*4*, *5*)
	SF3B4	-	
	SV2A	Body height	(*26*, *27*)
	BOLA1	-	
	HJV	-	
	POLR3GL	-	
	ANKRD34A	Leucocyte count	(*9*)
	RBM8A	-	
	LIX1L	-	
16	SPTLC3	-	
	TRERF1	Leucocyte count	(*4*, *10*, *14*)
	UBR2	-	
	CAMKMT	Body height	(*4*, *25*, *26*)
	TMEM247	-	
	EPAS1†	Erythrocyte count	(*4*)
		Hematocrit	(*10*)
		Hemoglobin measurement	(*10*, *28*)
		High altitude adaptation	(*29*, *30*)
		PR interval	(*31*)
	PRKCE†	Hematocrit	(*8*, *10*, *14*, *32*–*36*)
		Erythrocyte count	(*4*, *8*, *10*, *14*, *33*, *34*, *36*, *37*)
		Hemoglobin measurement	(*8*, *10*, *14*, *32*–*36*, *38*)
	MCFD2†	Hematocrit	(*10*, *14*)
		Erythrocyte count	(*10*, *14*)
		Hemoglobin measurement	(*10*, *14*, *38*)
		Body height	(*4*, *5*)
	LRFN2	BMI	(*4*, *12*, *39*)
38	NXPH1	-	
	GJD4‡	PR interval	(*40*)
	FZD8†	Hematocrit	(*8*)
		Erythrocyte count	(*8*, *10*)
		Hemoglobin measurement	(*4*, *10*)

Supplementary Figures

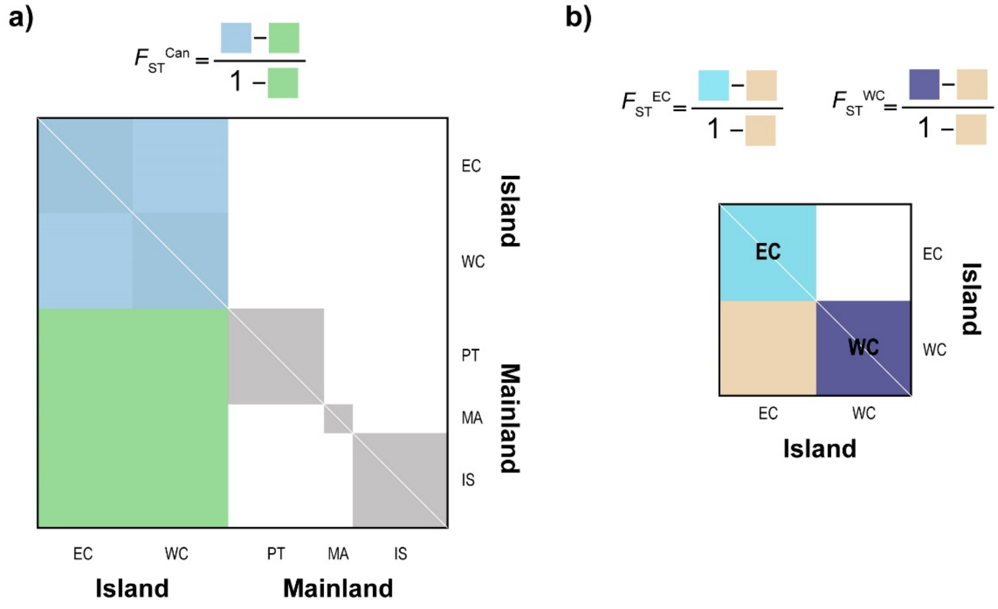

Sup. Fig. 1 – Graphical representation of the calculation of *F*β from individual relatedness matrices to compare **a)** insular and mainland individuals and **b)** individuals from each island. Coloured squares in formulas on top, represent the mean of the same-colour section of the matrix underneath, calculated in windows of 100kbp along the genome. Note that in **b)** the matrix is smaller as it does not include any owl from the mainland. In both matrices, the diagonal, in white, is empty.

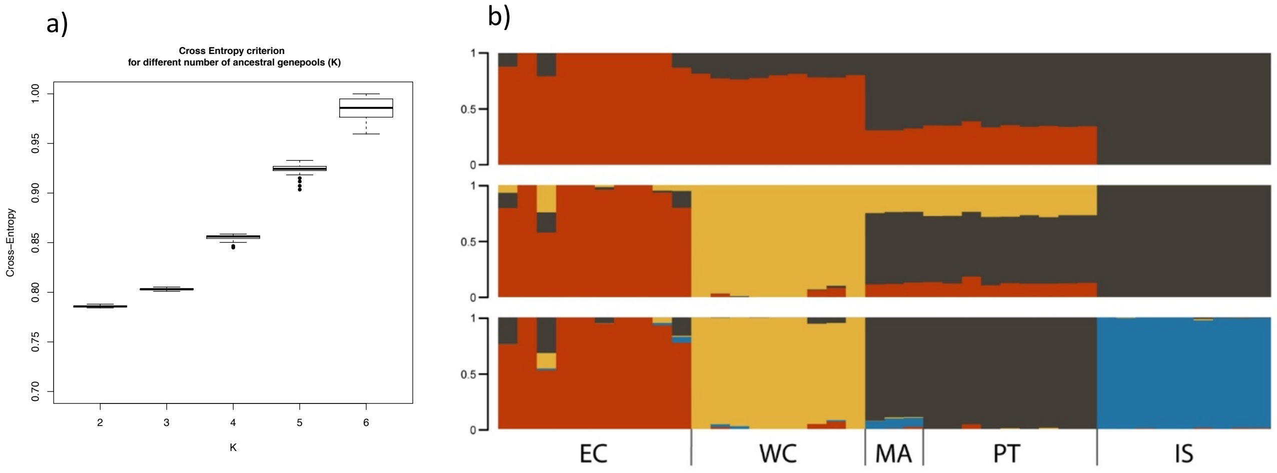

Sup. Fig. 2 – Individual barn owl clustering estimated by sNMF. **a)** Values of the cross-entropy criterion for 25 sNMF runs per K, with K varying between 2 and 6. The lower values suggest a better fit for K=2. **b)** Individual ancestry estimated for K ranging between 2 to 4. Each vertical bar represents one individual, and the colours represent the relative contributions of each genetic lineage.

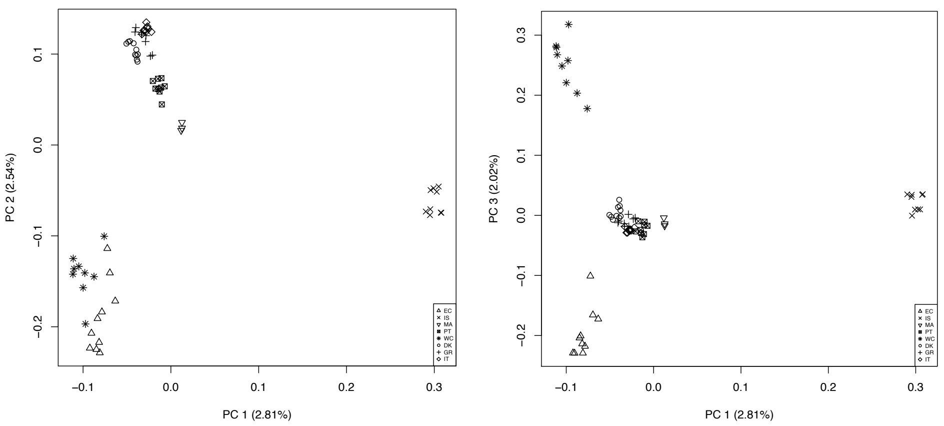

Sup. Fig. 3 – PCA of the barn owls used in this study and European ones from Cumer et al. (2021). Axes 1 to 3 of the PCA are depicted.

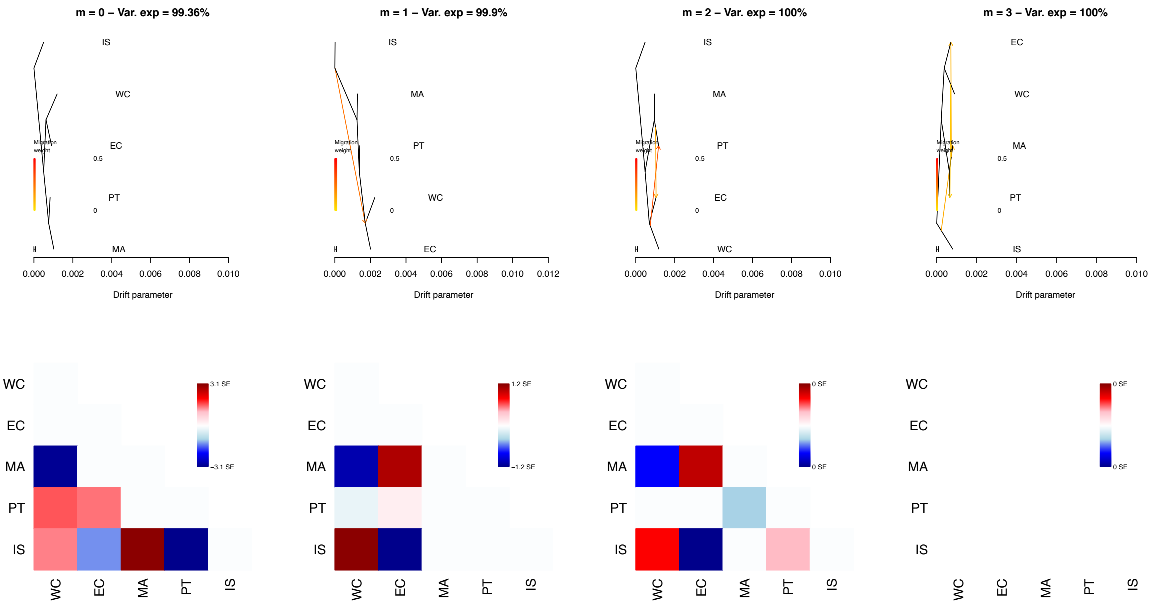

Sup. Fig. 4 – Treemix analysis with 0 and up to 3 migration events (m), the variance explained, and their residual matrices. The best runs of each migration are depicted. Note that for 2 & 3 migration events, the residual error is 0 and the variance explained 100%; treemix was thus unable to add more than one migration events to the tree.

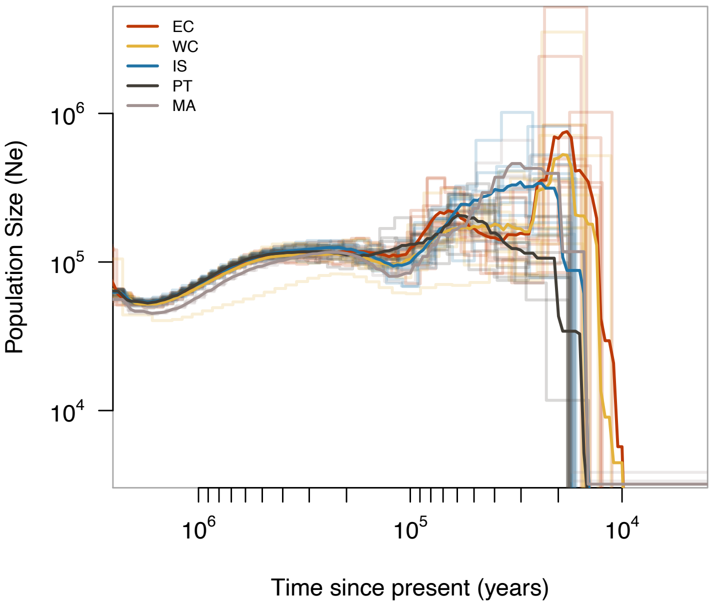

Sup. Fig. 5 – PSMC analysis. Inferred historical population sizes by pairwise sequential Markovian coalescent analysis. The x axis gives time scaled in years, assuming a mutation rate of 9.5^{-10} per site per year and a 2-year generation time. The y axis gives the effective population size measured by the scaled mutation rate.

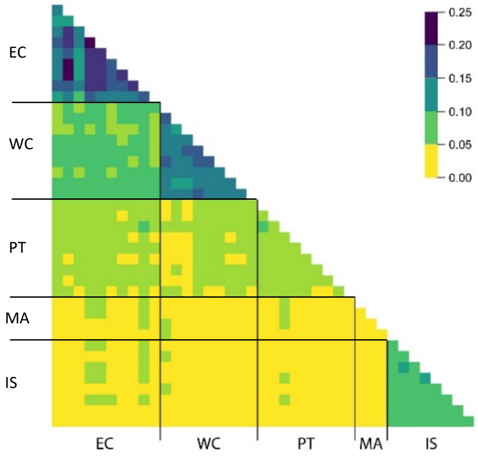

Sup. Fig. 6 – Pairwise individual relatedness (β) heatmap between all individuals.

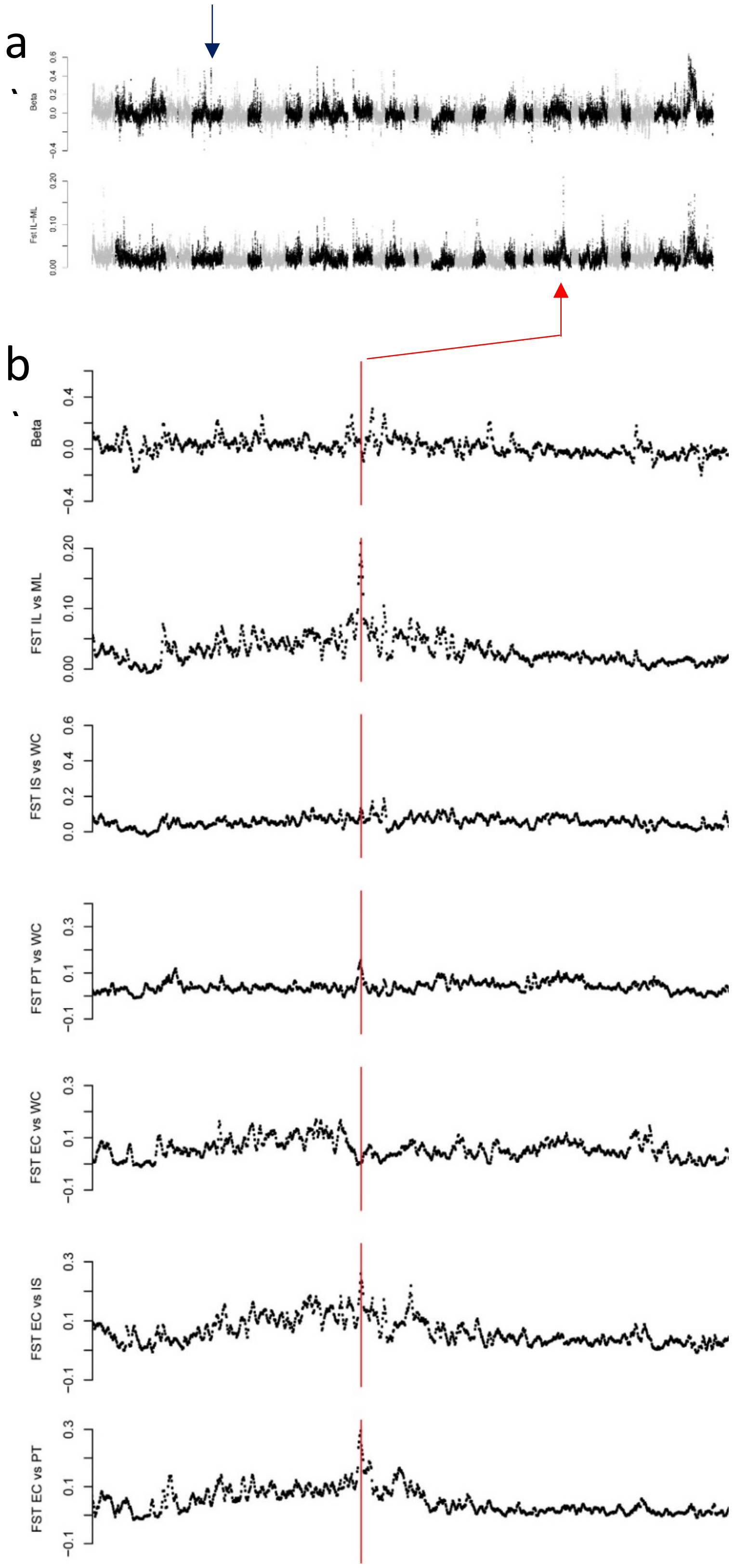

Sup. Fig. 7 – Comparison of F_{ST}^{Can} and F_{ST} scans for **a)** the whole genome and **b)** a zoom on the highest F_{ST}^{ILvsML} peak, absent from the F_{ST}^{Can} scan (indicated in red). Note that this peak is actually a specificity of EC and not an overall difference between insular and mainland owls. F_{ST}^{Can}

did not produce such a peak, instead identifying regions that were actually common to both islands in the comparisons to the mainland (example indicated in blue).

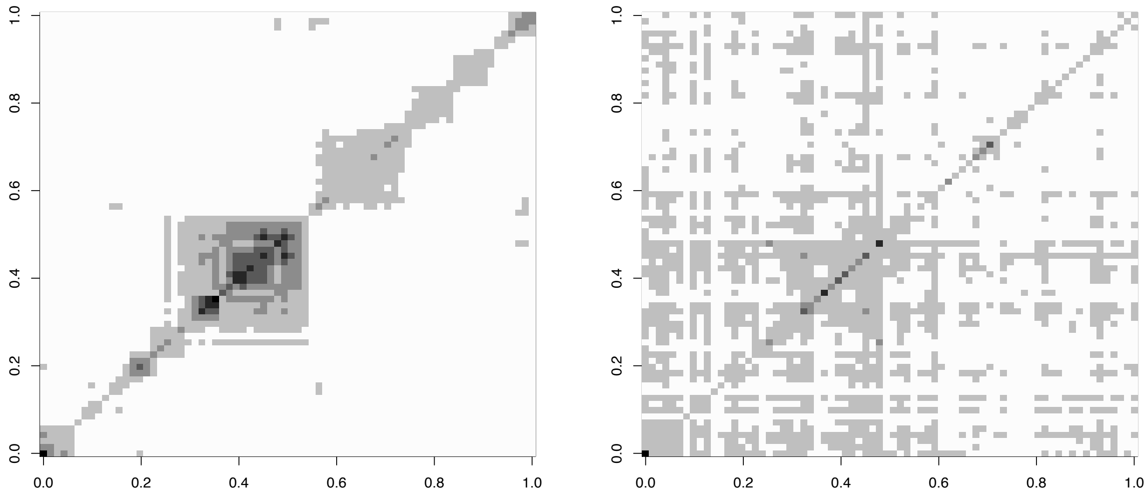

Sup. Fig. 8 – Matrices of pairwise Linkage Disequilibrium (LD; r^2) along Scaffold 10006 of barn owls from Islands and from Mainland. Each pixel shows the average r^2 of 100 SNP, with higher values being of darker colour. The Island matrix clearly shows a region of amplified LD that the Mainland does not. This region corresponds to the highly differentiated segment shown in Figure 2.

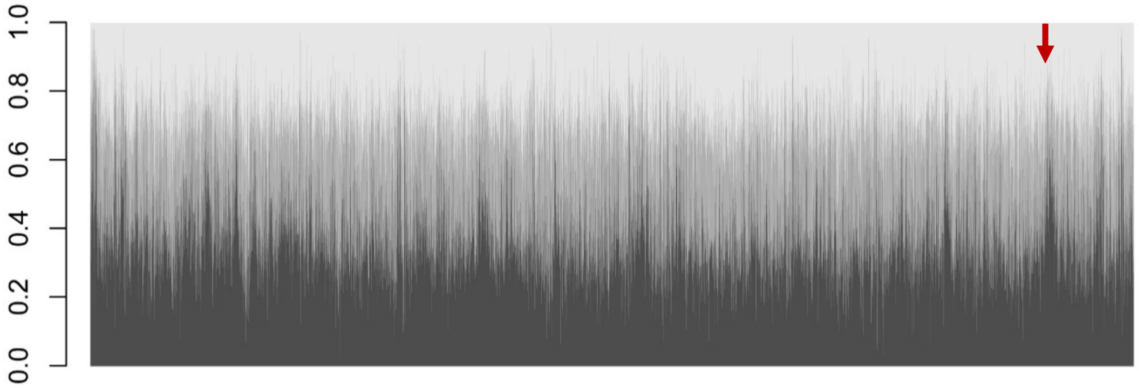

Sup. Fig. 9 – Twisst tree weighting output along the genome. At each 100kbp window, the proportion explained by each tree topology is shown in shades of grey (dark grey: ((WC,EC),PT,IS); grey: ((WC,PT),EC,IS); light grey: ((WC,IS),EC,PT)). Red arrow indicates the haplotype-like region selected on the islands (Figure 2). Note the increased proportion of dark grey trees, in which both island populations are placed in the terminal monophyletic branch, showing increased convergence among insular individuals in this genomic region.

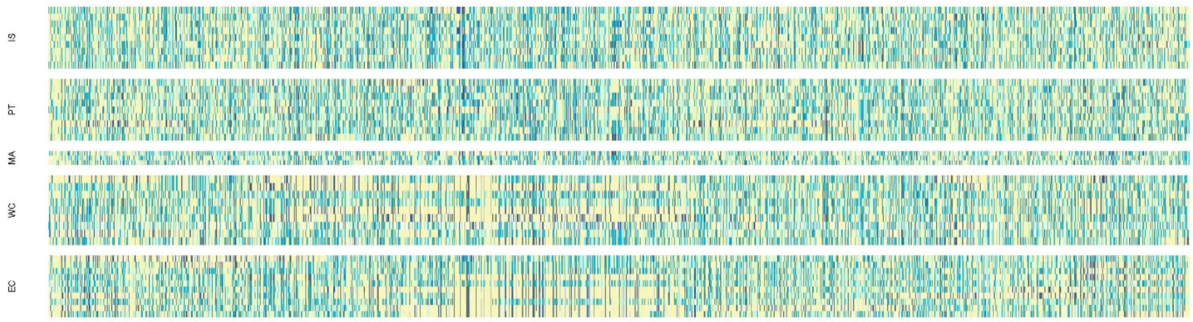

Sup. Fig. 10 – Allele dosage representation along Scaffold 10006 of barn owls from the Canary Islands and the Mediterranean Basin. Each vertical bar represents the genotype at one biallelic SNP. In yellow, homozygote for one allele; light blue, heterozygote; dark blue, homozygote for the other allele. The highly differentiated region shown in Figure 2 is visible with in the EC and to a lesser extend WC populations but not in the others, with clear long yellow segments.

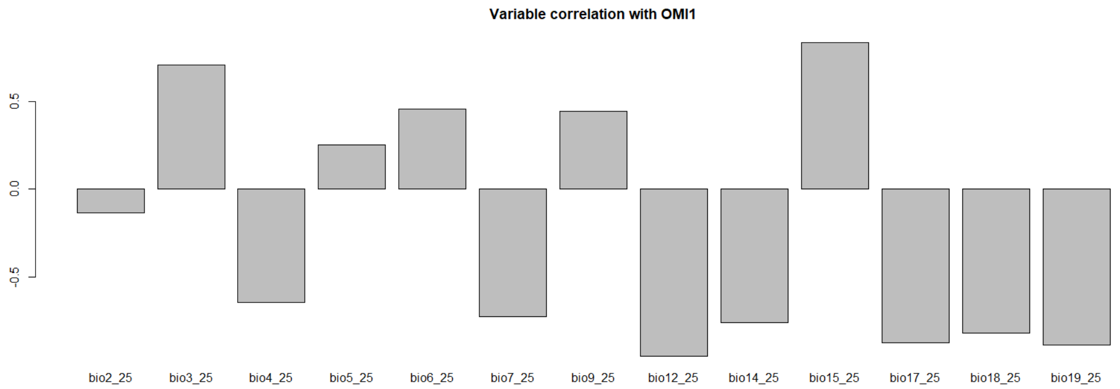

Sup. Fig. 11 – Correlation of bioclim climatic variables with OMI1, the first axis of niche variance.

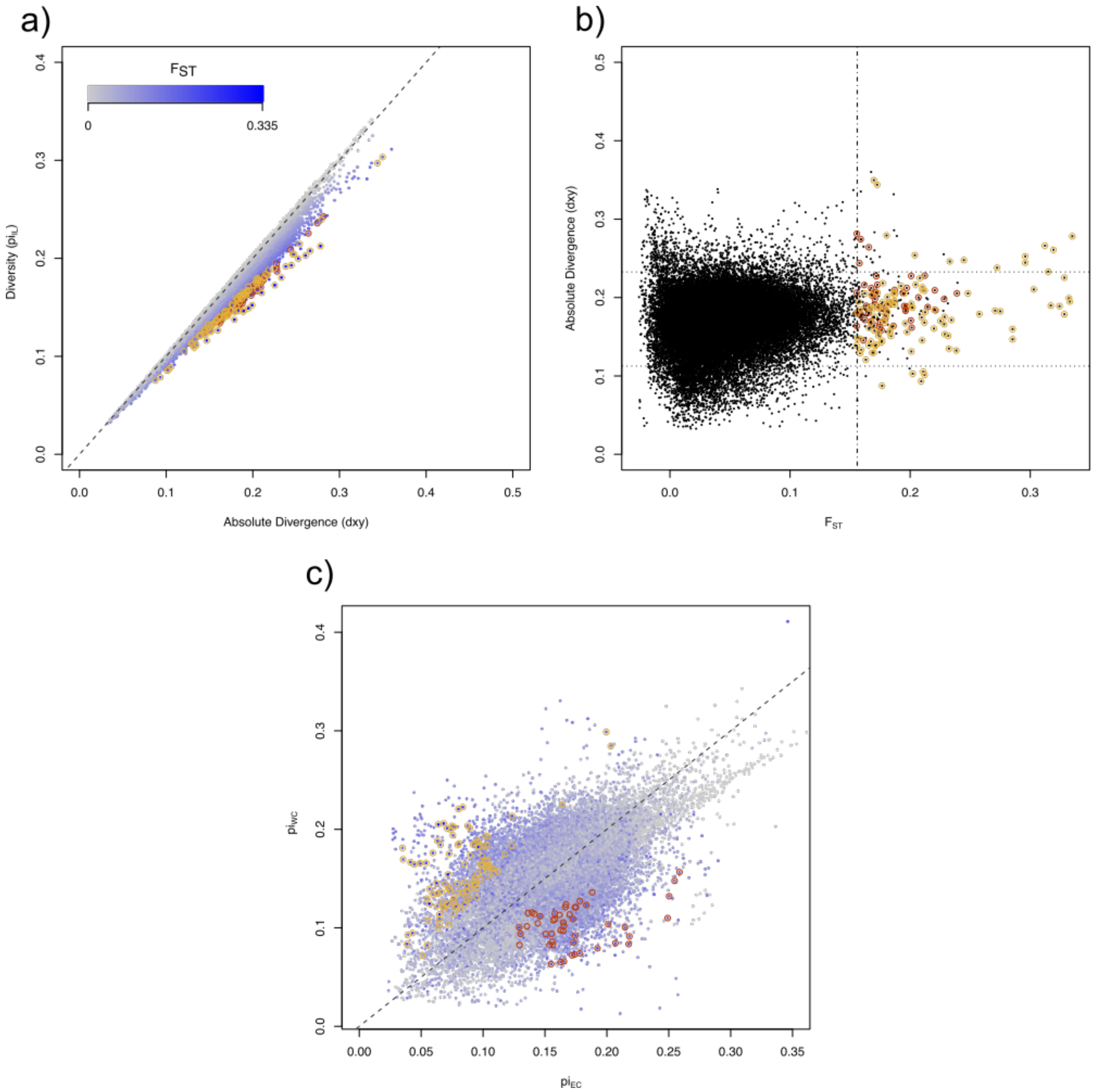

Sup. Fig. 12 – Correlation of different indexes in 100kbp sliding windows (20kbp slides) along the genome of the barn owl from the Canary Islands. **a)** Correlation between the absolute divergence (dxy) between the two islands populations (EC and WC) and the overall diversity present in the islands (π_{IL}). Color relate to pairwise FST value in the window, with bluer dots for higher values. Rounded dots highlight windows putatively under selection in each island population, with colours according to Figure 3. **b)** Correlation between pairwise F_{ST} between the two populations and the absolute divergence (dxy). Vertical line depicts the mean+5sd threshold used to detect regions putatively under selection based on the pairwise F_{ST}. Horizontal lines depict the mean+2sd of absolute divergence (dxy) between the two islands populations. **c)** Correlation between the diversity within each population (π_{EC} and π_{WC} for diversity within EC and WC population respectively). As in panel a), colours depict the pairwise FST value of the window, with bluer dots for higher values.

Supplementary References

1. T. Cumer *et al.*, *bioRxiv* (2021), doi:10.1101/2021.06.09.447652.
2. A. P. Machado *et al.*, *prep.*
3. A. P. Machado *et al.*, *bioRxiv* (2021), doi:10.1101/2021.04.23.441058.
4. G. Kichaev *et al.*, *Am. J. Hum. Genet.* **104**, 65–75 (2019).
5. I. Tachmazidou *et al.*, *Am. J. Hum. Genet.* **100**, 865–884 (2017).
6. S. P. Hagenaars *et al.*, *PLoS Genet.* **13**, e1006594 (2017).
7. M. Akiyama *et al.*, *Nat. Commun.* **10**, 1–11 (2019).
8. M. H. Chen *et al.*, *Cell*. **182**, 1198-1213.e14 (2020).
9. M. H. Chen *et al.*, *Cell*. **182**, 1198-1213.e14 (2020).
10. D. Vuckovic *et al.*, *Cell*. **182**, 1214-1231.e11 (2020).
11. V. Ramsuran *et al.*, *Clin. Infect. Dis.* **52**, 1248–1256 (2011).
12. Z. Zhu *et al.*, *J. Allergy Clin. Immunol.* **145**, 537–549 (2020).
13. V. Turcot *et al.*, *Nat. Genet.* **50**, 26–35 (2018).
14. W. J. Astle *et al.*, *Cell*. **167**, 1415-1429.e19 (2016).
15. A. Giri *et al.*, *Nat. Genet.* **51**, 51–62 (2019).
16. E. Evangelou *et al.*, *Nat. Genet.* **50**, 1412–1425 (2018).
17. A. M. Kulminski *et al.*, *Aging (Albany. NY).* **10**, 492–514 (2018).
18. T. J. Hoffmann *et al.*, *Nat. Genet.* **49**, 54–64 (2017).
19. C. Liu *et al.*, *Nat. Genet.* **48**, 1162–1170 (2016).
20. L. V. Wain *et al.*, *Hypertension*. **70**, e4–e19 (2017).
21. G. B. Ehret *et al.*, *Nat. Genet.* **48**, 1171–1184 (2016).
22. C. Newton-Cheh *et al.*, *Nat. Genet.* **41**, 666–676 (2009).
23. P. Surendran *et al.*, *Nat. Genet.* **48**, 1151–1161 (2016).
24. C. A. German, J. S. Sinsheimer, Y. C. Klimentidis, H. Zhou, J. J. Zhou, *Genet. Epidemiol.* **44**, 248–260 (2020).
25. A. R. Wood *et al.*, *Nat. Genet.* **46**, 1173–1186 (2014).
26. H. L. Allen *et al.*, *Nature*. **467**, 832–838 (2010).
27. D. F. Gudbjartsson *et al.*, *Nat. Genet.* **40**, 609–615 (2008).
28. C. Jeong *et al.*, *PLoS Genet.* **14**, e1007650 (2018).
29. C. Jeong *et al.*, *Nat. Commun.* **5** (2014), doi:10.1038/ncomms4281.
30. J. Yang *et al.*, *Proc. Natl. Acad. Sci. U. S. A.* **114**, 4189–4194 (2017).
31. I. Ntalla *et al.*, *Nat. Commun.* **11**, 1–12 (2020).
32. S. K. Ganesh *et al.*, *Nat. Genet.* **41**, 1191–1198 (2009).
33. M. Kanai *et al.*, *Nat. Genet.* **50**, 390–400 (2018).
34. F. J. A. van Rooij *et al.*, *Am. J. Hum. Genet.* **100**, 51–63 (2017).
35. M. H. Kowalski *et al.*, *PLoS Genet.* **15**, e1008500 (2019).
36. C. J. Hodonsky *et al.*, *PLoS Genet.* **13**, e1006760 (2017).
37. Y. Kamatani *et al.*, *Nat. Genet.* **42**, 210–215 (2010).
38. G. R. Oskarsson *et al.*, *Commun. Biol.* **3**, 1–10 (2020).
39. S. L. Pulit *et al.*, *Hum. Mol. Genet.* **28**, 166–174 (2019).
40. X. Deng *et al.*, *PLoS One*. **8**, e79629 (2013).
